# Examining the molecular basis of coat color in a nocturnal primate family (Lorisidae)

**DOI:** 10.1002/ece3.7338

**Published:** 2021-03-10

**Authors:** Rachel A. Munds, Chelsea L. Titus, Lais A. A. Moreira, Lori S. Eggert, Gregory E. Blomquist

**Affiliations:** ^1^ Department of Anthropology & Archaeology University of Calgary Calgary AB Canada; ^2^ Nocturnal Primate Research Group Oxford Brookes University Oxford UK; ^3^ Division of Biological Sciences University of Missouri Columbia MO USA; ^4^ Department of Anthropology University of Missouri Columbia MO USA

**Keywords:** aposematic, deleterious, *MC1R*, nonsynonymous, parallel evolution, pigmentation, purifying selection

## Abstract

Organisms use color for camouflage, sexual signaling, or as a warning sign of danger. Primates are one of the most vibrantly colored Orders of mammals. However, the genetics underlying their coat color are poorly known, limiting our ability to study molecular aspects of its evolution. The role of the melanocortin 1 receptor (*MC1R*) in color evolution has been implicated in studies on rocket pocket mice (*Chaetodipus intermediusi*), toucans (Ramphastidae), and many domesticated animals. From these studies, we know that changes in *MC1R* result in a yellow/red or a brown/black morphology. Here, we investigate the evolution of *MC1R* in Lorisidae, a monophyletic nocturnal primate family, with some genera displaying high contrast variation in color patterns and other genera being monochromatic. Even more unique, the Lorisidae family has the only venomous primate: the slow loris (*Nycticebus*). Research has suggested that the contrasting coat patterns of slow lorises are aposematic signals for their venom. If so, we predict the *MC1R* in slow lorises will be under positive selection. In our study, we found that Lorisidae *MC1R* is under purifying selection (*ω* = 0.0912). In Lorisidae *MC1R,* there were a total of 75 variable nucleotides, 18 of which were nonsynonymous. Six of these nonsynonymous substitutions were found on the *Perodicticus* branch, which our reconstructions found to be the only member of Lorisidae that has predominantly lighter coat color; no substitutions were associated with *Nycticebus*. Our findings generate new insight into the genetics of pelage color and evolution among a unique group of nocturnal mammals and suggest putative underpinnings of monochromatic color evolution in the *Perodicticus* lineage.

## Graphical abstract

In primates, the most colorful of mammals, few have examined the influence the melanocortin 1 receptor (*MC1R*) has on color variation (light or dark) in primates. Here, we examined how *MC1R* impacts color variation in a cryptic primate family with high contrasting color variation. We found *MC1R* is under purifying selection, but there appears to be a link in nucleotide changes and light coat color.

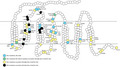

## 1 INTRODUCTION

The evolution of color variation in organisms highlights the selective pressures that impact survivability. Research on color variation has demonstrated coloration contributes to the ability of animals to conceal themselves from predators, to regulate body temperatures, to advertise their toxicity, or to attract mates (c.f. Caro, 2005). For taxonomists, striking color differences and patterns have been used for distinguishing species (Bradley & Mundy, 2008; Caro, 2009; Nekaris & Jaffe, 2007). Several genetic loci have been identified that influence coat color and pattern variation in numerous organisms, but the most well‐studied locus in vertebrates is the melanocortin 1 receptor (*MC1R*).

The *MC1R* is involved in the regulation of the melanin production of two common pigments: eumelanin (brown and black) and pheomelanin (yellow and red) (Barsh, 1996; Hoekstra & Nachman, 2003). Studies on *MC1R* variation have focused on differences between melanistic, or dark‐colored (brown/black) morphs, and amelanistic, or light‐colored morphs (yellow/red). For ease of understanding, we will refer to those with a melanistic phenotype as “dark” and those with an amelanistic phenotype as “light.” Changes in *MC1R* have been associated with color changes in insects, birds, mice, domesticated livestock and pets, and other mammals (Theron). Many of these studies examined the synonymous (d*S*: a substitution that does not change the amino acid structure) and nonsynonymous (d*N*: a substitution that changes the amino acid structure) substitution ratio in *MC1R*. The d*S*/d*N* ratio is used as an indicator of the strength and mode of natural selection in a coding gene over an extended timescale. A d*S*/d*N* ratio of less than 1 is associated with purifying selection, equal to 1 is neutral selection, and greater than 1 is positive selection (Yang & Bielawski, 2000). Generally, the d*S*/d*N* ratio is expected to be less than 1 in coding regions as changes in the amino acid structure in coding genes can be detrimental, thus resulting in fewer nonsynonymous substitutions (Jeffares et al., 2015). But positive selection can occur on coding genes if changes are beneficial.

As stated, *MC1R* influences vertebrate pigmentation (Barsh, 1996; Hoekstra, 2006; Mundy, 2005; Protas & Patel, 2008; Rees, 2003; Theron et al., 2001), but it is not the only gene to influence coat or hair color differences, as the agouti signaling protein, encoded by the *ASIP* gene, is another well‐studied locus that regulates the distribution of melanin in mammals. Changes in *ASIP* result in agouti coloration: single hair bands with alternating yellow and black sections of varying size (Fontanesi et al., 2009; Furumura et al., 1996). Other key pigmentation genes have been identified and research on this topic is growing, but much of it is focused on domesticated animals, like livestock and dogs, and mice—predominantly laboratory mice (Daverio et al., 2016; Mallarino et al., 2016; Neves et al., 2017; Protas & Patel, 2008; Rees, 2003; Saif et al., 2020; Silvers, 2012; Yang et al., 2019; Zhao et al., 2018). Although these studies provide invaluable information, their methods and results can be difficult to transfer to wild, less‐studied populations. For example, the ALX homeobox 3 (*ALX3*) in African striped mice (*Rhabomys pumilo*) impacts dorsal stripe formation, and *ASIP* contributes to the shade of brown produced in these stripes (Mallarino et al., 2016). Mutations of the KIT proto‐oncogene, receptor tyrosine kinase (*KIT*), have been noted in mice, horses, cats, and other vertebrates and are attributed to white morphs as well as white spotting that can result in “masking” patterns on the face and spotting patterns throughout the body (Brookes & Bailey, 2005; David et al., 2014; De Sepulveda et al., 1995; Wong et al., 2013). In dogs, melanocyte‐inducing transcription factor (*MITF*) is known to contribute to irregular white spotting, and numerous other canid genes have been identified that result in their color and pattern differences (Saif et al., 2020). Yet, few studies have examined these genes in wild populations, or explored the role of *MC1R* in color regulation in nondomesticated species, such as primates (c.f. Mundy & Kelly, 2003). Even our own study attempted to examine *KIT* and *ASIP* in nonhuman primates, but we were unable to design successful primers for amplification based on available primers from closely related species (Mundy & Kelly, 2006) or published sequences. These regions of the genome could be different enough in Lorisidae that it made it challenging to successfully amplify them. Establishing a baseline of color evolution in a group of organisms will aid in the development of future projects to examine color variation, which is easiest to do by using the most well‐studied and understood pigmentation gene: *MC1R*.

For example, Nachman et al. (2003) found four adaptive charge‐changing amino acid polymorphisms in the *MC1R* of rock pocket mice (*Chaetodipus intermediusi*) in Arizona. These *MC1R* amino acid changes were linked to aiding the mice in blending in with their environment. Mice living in light‐colored environments had substitutions associated with light‐color phenotypes and vice versa for dark‐colored mice (Nachman et al., 2003). Corso et al. (2016) examined variation in *MC1R* sequences in toucans (Ramphastidae). By comparing the dS/dN ratio in *MC1R* and relating it to the observed phenotypic changes, they were able to demonstrate that molecular genetic variants were related to the evolution of feather coloration in Ramphastidae. For toucans, they suggested that darker plumage resulted from either positive selection or a relaxation of selection on *MC1R* (Corso et al., 2016). In humans (*Homo sapiens*), changes in *MC1R* are linked to hair and skin color variation. Lighter skin pigmentation, red/yellow hair color, freckles, and sensitivity to UV are the result of more than 30 nonsynonymous mutations in the *MC1R* gene (Sulem et al., 2007). The changes in hair color production are due to a relationship between *MC1R* and *ASIP* (Beaumont et al., 2007; Harding et al., 2000; Hoekstra, 2006; Moro et al., 1999; Mundy & Kelly, 2003, 2006; Nasti & Timares, 2015; Valverde et al., 1995). The transition to the production of pheomelanin is considered to be derived, whereas the production of eumelanin is ancestral (Han et al., 2008; Jackson, 1997; Sturm et al., 2003; Sulem et al., 2007). These studies and the extensive body of literature on *MC1R* demonstrate that just one amino acid coding change in *MC1R* can result in stark color differences among closely related taxa and may be the product of selection (Corso et al., 2016; Gompel & Prud'homme, 2009; Hoekstra, 2006; Majerus & Mundy, 2003; Nunes et al., 2011).

Less studied is the impact of *MC1R* mutations on nonhuman primates, even though nonhuman primates are considered to be some of the most colorful and patterned mammals (Bradley & Mundy, 2008; Moreira et al., 2019). One of the first and seminal studies of *MC1R* in nonhuman primates found that nonsynonymous mutations were not correlated to coat color and appeared to be more likely influenced by phylogeny (Mundy & Kelly, 2003). This study also found that purifying selection is the primary mode of evolution for the *MC1R* gene in nonhuman primates. There were a few exceptions, especially with respect to *Leontopithecus rosalia* (golden lion tamarin), where results indicated a higher‐than‐expected d*N*/d*S* ratio (0.91) that was not significantly different from 1 and revealed several substitutions and deletions at functionally important sites. Their finding suggests the red hair phenotype observed in *L. rosalia* could be from a loss of function in *MC1R*, as found in humans (Mundy & Kelly, 2003). Although their study is one of the most comprehensive studies of *MC1R* evolution in nonhuman primates, Mundy and Kelly (2003) focused mainly on monkeys and apes (catarrhines). Another study examined functional changes within *MC1R* in nine phylogenetically distant primate species with varying coat colors (Haitina et al., 2007). Although this study found an amino acid substitution (E94K) that corresponds to color change in various vertebrates, this particular substitution does not seem to affect color change in the studied primates. Other substitutions were noted that possibly impact ligand binding sites, such as D117G and D121N. Additionally, their examination of the red/orange orangutans found no *MC1R* mutations associated with a pure pheomelanin coat production; not even substitutions similar to ones found in humans (Haitina et al., 2007). As for studies of *MC1R* on a finer scale, there have been two studies that examined *MC1R* variation in and among macaque species (*Macaca*) (Bradley et al., 2013; Nakayama et al., 2008). Neither study was able to find support for *MC1R* to be influencing coat color changes within these species, suggesting *MC1R* may not be the ideal gene to use when trying to infer coat color genetics or evolution in catarrhine primates. Neither study examined *MC1R* evolution and its association with skin color. Furthermore, no studies have solely focused on *MC1R* mutations in lemurs or lorises (strepsirrhines). Studies that did include strepsirrhines either treated them as an outgroup to catarrhines or found little support for an effect of change within *MC1R* on coat color (Mundy & Kelly, 2003).

However, coat coloration might not play the same role in catarrhine and diurnal species as it does in nocturnal species. For nocturnal primates, the contrasting light and dark coloration is essential for visual recognition at night, and aids in intraspecific identification and communication, as well as camouflage (Bearder, 1999; Caro, 2005; Ford, 1994; Munds et al., 2013; Nekaris & Bearder, 2007). The dark coloration can result in different contrasting values, which further reinforces species diversification (Ford, 1994; Munds et al., 2013). Within the nocturnal primate family Lorisidae (*Nycticebus*, *Loris*, *Perodicticus*, *Arctocebus*), variation might not just be reflective of conspecific recognition, it might also be aposematic signaling. The slow loris (*Nycticebus*) is the only venomous primate (Nekaris et al., 2013). This has led some to propose that their coat patterns and coloration are aposematic signals, warning predators, and conspecifics of their dangerous venom, as well as a form of mimicry to the sympatric cobras (c.f. Nekaris et al., 2013).

Aposematism is as a type of interspecific signal that warns a predator this particular prey is noxious. These signals can be bright colors or, as typically observed in mammals, distinct contrasting markings such as the stripes found on skunks (Caro, 2009, 2013). Slow lorises possess distinct contrasting dorsal stripes and facemasks, and when threatened, they apply a toxin secreted from glands in the armpits to their mouth. The mixing of this toxin with saliva makes the venom. After application, the slow loris will then connect the hands above its head, in an appearance that is like the fanning of a cobra‐hood. The ocular patches of the loris with the raised arms appear to replicate the open hood of an Indian cobra (*Naja naja*) with its two distinct spots (Nekaris et al., 2013). Thus, the slow loris could be a Mullerian mimic (when a toxic animal imitates or appears like another toxic animal) through behavior and replication of the spots (Mallet & Joron, 1999; Pasteur, 1982). Of even more interest is that slender lorises are not venomous but they display contrasting ocular patches and when threatened will raise their arms over their heads like slow lorises (Gursky‐Doyen & Nekaris, 2007). Such behavior and markings suggest slender lorises are Batesian mimics (a nontoxic animal that takes on the appearance and or behavior of a toxic animal) of cobras and/or slow lorises (Mallet & Joron, 1999; Pasteur, 1982). The evolution of aposematic coloration and patterning is speculated to be selected upon and seems to result from a few loci changes, such as one or two changes within *MC1R*, that result in major phenotypic changes (Mallet & Joron, 1999). If this is the case for Lorisinae, then we would expect them to have *MC1R* nonsynonymous mutations on their branch in comparison with the closely related African subfamily Perodictinae (*Perodicticus*, *Arctocebus*), who are monochromatic, lack venom, or mimetic. Such dichotomous coat colors and patterns coupled with one subfamily evolving rare defense strategies (venom and mimicry) not seen in any other primate present a unique opportunity to examine the evolution of coat color and its association with aposematic/mimicry and phylogenetic signals.

**FIGURE 1:**
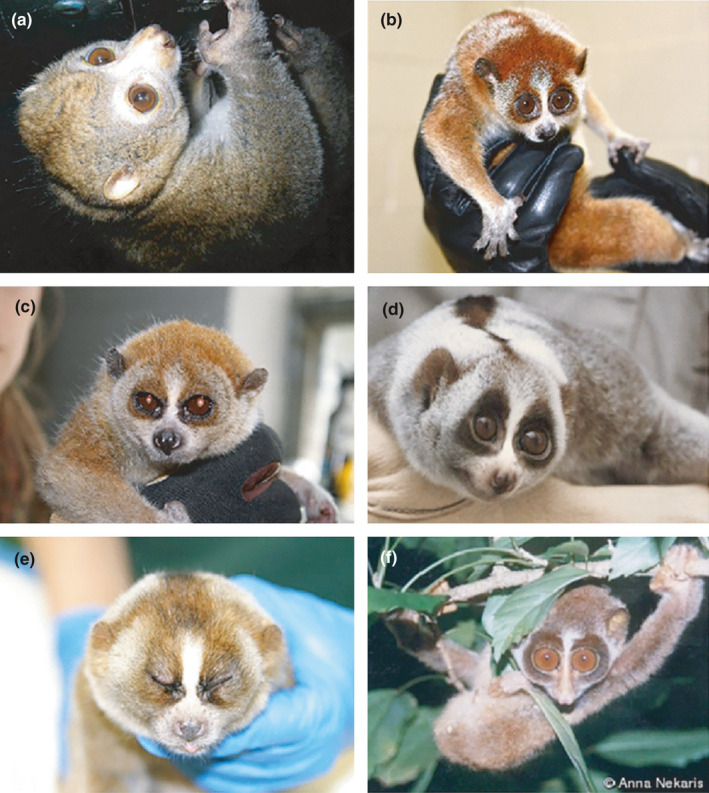
Coat colors and patterns of examined Lorisidae species: (a) *Perodicticus potto* (Cincinnati Zoo), (b) *Nycticebus pygmaeus* (Brookfield Zoo, Chicago), (c) *N. pygmaeus* (Capron Zoo), (d) *N. coucang* (possible *N. bengalensis*) (San Diego Zoo/courtesy Dr. Helena Fitch‐Snyder), (e) *N. coucang* (Minnesota Zoo), and (f) *Loris tardigradus* (image copyright Dr. K.A.I. Nekaris)

We predict that Perodictinae, more specifically *Perodicticus*, retain the ancestral form of Lorisidae coloration due to their monochromatic phenotype and lack of specialized defenses. We expect the overall Lorisidae d*N*/d*S* ratio of *MC1R* to indicate purifying selection (e.g., <1), as has been found with numerous other studies, including the few that examined primates (Majerus & Mundy, 2003; Makova & Norton, 2005; Mundy & Kelly, 2003; Nunes et al., 2011). Such a finding would not suggest *MC1R* lacks a functional role in coat or skin morphology in Lorisidae, but instead would indicate it is conserved as changes in *MC1R* affect survival (Beaumont et al., 2007; Harding et al., 2000; Hoekstra, 2006; Makova & Norton, 2005; Moro et al., 1999; Nasti & Timares, 2015; Valverde et al., 1995). Finally, we predict there are more nonsynonymous mutations along the Lorisinae (*Loris* and *Nycticebus*) branches than along the Perodictinae branches (*Arctocebus* and *Perodicticus*). Our predictions are based on the idea that the color and patterning found in Lorisinae are adaptive (Figure 1).

## 2 MATERIAL AND METHODS

### 2.1 Study collection

Due to the difficulty of trapping Lorisidae from the wild (Munds et al., 2018; Nekaris et al., 2020), we obtained samples (already extracted DNA from various tissue sources, or whole tissue samples we extracted from such as hair) and photographs from American Zoological Association (AZA) institutions. As the majority of our tissue samples are from hair follicles which are a poor source of genomic DNA, we focused our project on a few key genes (*MC1R*, mitochondrially encoded cytochrome b (*MT‐CYB*)), rather than taking a more genome‐wide approach. Additionally, collecting from captive institutions limits the species studied as only two to three of the recognized eight slow loris species are found in captivity (*N. coucang*, *N. bengalensis*, *N. pygmaeus*), possibly two species of slender loris (*L*. *tardigradus*, *L. lydekkerianus*), and only one member of the subfamily Perodictinae, but the specific species or subspecies is unknown (*Perodicticus*). Currently, there are three recognized species of *Perodicticus*, but it is uncertain which species are represented in captivity. There is a good probability that captive individuals are hybrids, as the new species are recently recognized and all species are highly similar in appearance (Mittermeier et al., 2013). The genus *Arctocebus* is not found in captivity and thus could not be included in our study. As there are multiple species of *Nycticebus* species in captivity, we will refer to it as *Nycticebus* spp. or *N*. spp., except in regard to *N. pygmaeus* which we will keep separate as studies have determined *N. pygmaeus* to be morphologically and genetically distinct from other *Nycticebus* species (Munds et al., 2018; Nekaris & Munds, 2010; Pozzi et al., 2015).

### 2.2 Photography collection

**FIGURE 2:**
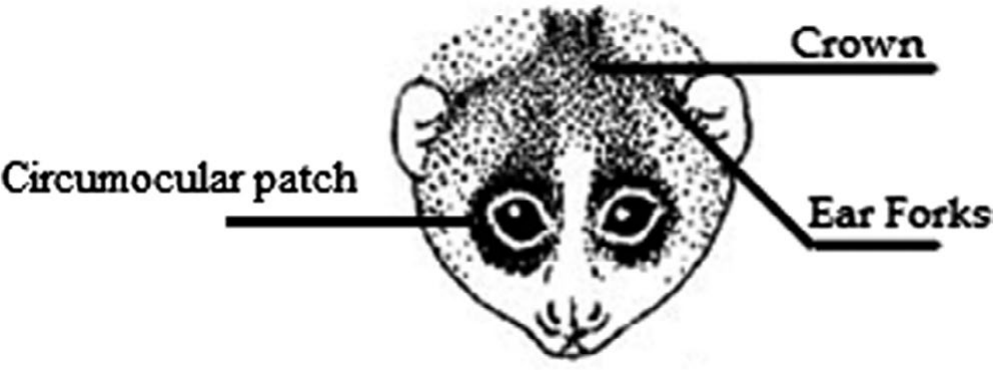
Hair features examined for color identification. Drawing by H. Schulze

We requested photographs of all genetically sampled individuals from participating AZA institutions, with four photographs per animal. Specifically, we requested each animal have left side and full frontal of the face, top of the head, and the entire dorsal photographed (Figure 2). Some of the genetic samples used were acquired from deceased Lorisidae (Frozen Zoo^®^ and Duke Lemur Center (DLC)), which limited the number of photographs we had for these individuals. With *Nycticebus* from the Frozen Zoo^®^ collection, we were able to get all four photographs requested from historical records. DLC shared a few photographs of the two *N. pygmaeus* individuals we examined from their institution. We were not able to obtain photographs of the slender lorises (*Loris*) used in this study but acquired photographs from other zoo websites (Memphis Zoo, San Diego Zoo, London Zoo, Antwerp Zoo, and Biblical Gardens). We cannot confirm the relationship of these captive individuals to our genetics samples, but many captive individuals are related to each other, so using photographs from these other slender lorises still provides an accurate depiction of slender loris coat and skin colors. Additionally, although there are species differences between the gray and red slender lorises, individual differences are considered to be minimal within species (Nekaris & Bearder, 2007). The photographs shared by AZA institutions are not our property and cannot be made publicly available. We are happy to share images privately, with approval from the AZA institutions.

### 2.3 Ancestral state reconstruction

We analyzed one to six images from faces and bodies of 28 individual Lorisidae (4 *Perodicticus*, 11 *N. pygmaeus*, 4 *Nycticebus* spp., and 7 *Loris*). Additionally, we included to our analyses nonloris outgroups: 30 members of three species of lemurs (10 *Eulemur fulvus fulvus*, 10 *Varecia rubra*, and *10 Varecia variegata*), 10 individuals to represent the family Galagidae (*Galago senegalensis*), 20 Callitrichidae (10 *Leontopithecus rosalia* and 10 *Callimico goeldii*), and 20 individuals of the Cercopithecidae family (10 *Macaca mulatta* and 10 *Macaca nigra*). To increase the number of images analyzed, we selected photographs from online image libraries (https://commons.wikimedia.org; https://www.biolib.cz;) (Supplemental Material S1). It is important to note that while we carefully chose the photographs from different online image libraries, there could be some pseudoreplications among them. However, we do not expect pseudoreplications to interfere with our results given our large sample size per species. Features analyzed included facial and body areas predominantly covered with hair (circumocular patch, crown, ear forks, dorsal stripe, and rump) and facial areas that have skin completely exposed (nose, ear, hand, and around the eyes). It is important to note that some of these areas (i.e., circumocular patch, crown, ear forks, and dorsal stripe) are not present in all species examined. Nevertheless, we carefully selected areas that are functionally like the ones in Lorisidae. The analyzed features were previously shown to provide meaningful discrimination between slow loris species (Munds et al., 2013; Nekaris & Jaffe, 2007; Nekaris & Munds, 2010). The skin area predominantly covered with hair (hereafter, hair) and completely exposed (hereafter, skin) were subjectively categorized as white, yellow, red, orange, brown, black, gray, and pink (Supplemental Material S2). The colors of facial and body areas were independently coded by three raters. Because coding colors is subjective, we measured the inter‐rater agreement by using the Fleiss' kappa test (Fleiss, 1971; Fleiss et al., 2003) and our results indicated that the strength of agreement between observers was good (*K* = 0.62; *p* < .005). We should point out that while the strength of agreement was found to be sufficient (Altmann, 1999; Arstein & Poesio, 2008), kappa values lower than 0.8 have been found to introduce some degree of noise to the data. Our kappa values are below this threshold, but we feel confident in our results, given the general agreement between individuals in assigning color values. The answers were compared and the most common answer to a trait was used in the analysis.

We performed ancestral state reconstructions by mapping the hair and skin colors using a phylogenetic tree drawn from a published Lorisidae phylogeny (Arnold et al., 2010; Munds et al., 2018). We inferred the evolutionary history of hair and skin color (discrete traits) using a stochastic mapping approach implemented in the R phytools package (Revell, 2013). The ancestral states at each node were estimated under three basic models: equal rates (ER), all rates different (ARD), and symmetrical transition rate (SYM). The ER was the best fitting model. From this model, we simulated 1,000 character histories across the phylogeny using the phytools make.simmap function in R version 3.6.3 (Bollback, 2006).

### 2.4 Tissue collection and sequencing

The collection protocol adhered to the humane animal handling guidelines (The Animal Behavior Society, 2003), and samples were collected by trained personnel at each institution. To reduce the risk of contamination, handlers were instructed to wear sterile gloves and to use a piece of masking tape to remove the ~20 hair and follicles from the dorsal neck area of individual lorises. The use of tape for tissue collection is fast, reduced handling time, cost‐effective, and does not require sterilization between individuals. Our results from this study found no contamination issues with this method. Each sample was stored separately in a clean, dry coin envelope. Additionally, the Duke Lemur Center provided liver samples from two deceased *N. pygmaeus* individuals, and prepared extracted DNA was provided from the Frozen Zoo^®^ collection at the San Diego Institute for Conservation Research. From San Diego, we received 5 *N*. spp., 1 *N. pygmaeus*, and 4 *Loris* samples. In total, we analyzed samples from 24 Lorisidae individuals (Table 1).

**TABLE 1:** GenBank sequences and Lorisidae samples used in this study

Genus/Species	Identification	Specimen facility	Sample type
*Nycticebus* spp.	SD734	San Diego Zoo	DNA
	SD283	San Diego Zoo	DNA
	SD303	San Diego Zoo	DNA
	SD435	San Diego Zoo	DNA
	SD302	San Diego Zoo	DNA
	MZ9750	Minnesota Zoo	Hair
	MZ9585	Minnesota Zoo	Hair
*Nycticebus pygmaeus*	DLC001	Duke Lemur Center	Tissue
	DLC002	Duke Lemur Center	Tissue
	CZM1	Capron Park Zoo	Hair
	SD299	San Diego Zoo	DNA
	CZSC1	Chicago Zoological Society	Hair
	ABQP1	ABQ BioPark	Hair
	CZBGC1	Cincinnati Zoo & Botanical Garden	Hair
*Loris*	SD699	San Diego Zoo	DNA
	SD138	San Diego Zoo	DNA
	SD698	San Diego Zoo	DNA
	SD203	San Diego Zoo	DNA
*Perodicticus*	CZBGH1	Cincinnati Zoo & Botanical Garden	Hair
	CZBGM1	Cincinnati Zoo & Botanical Garden	Hair
	CZBGG1	Cincinnati Zoo & Botanical Garden	Hair
	CZBGJ1	Cincinnati Zoo & Botanical Garden	Hair
	CZBGI1	Cincinnati Zoo & Botanical Garden	Hair
	CMPT1	Cleveland Metroparks Zoo	Hair
*Callimico*	AY205121.1	GenBank	*MC1R*
	KR528428.1	GenBank	*MT‐CYB*
*Eulemur*	AY205141.1	GenBank	*MC1R*
	AF081050.1	GenBank	*MT‐CYB*
*Galago*	AY205138.1	GenBank	*MC1R*
	AY441470.1	GenBank	*MT‐CYB*
*Leontopithecus*	AY205123.1	GenBank	*MC1R*
*rosalia*	KR528404.1	GenBank	*MT‐CYB*
*Macaca mulatta*	AB296173.1	GenBank	*MC1R*
	U38272.1	GenBank	*MT‐CYB*
*Macaca nigra*	AB296208.1	GenBank	*MC1R*
	AF350387.1	GenBank	*MT‐CYB*
*Varecia rubra*	AY205139.1	GenBank	*MC1R*
	U53578.1	GenBank	*MT‐CYB*
*Varecia variegata*	AY205140.1	GenBank	*MC1R*
	AF081047.1	GenBank	*MT‐CYB*

DNA hair follicle extraction followed the InstaGene protocol from Eggert et al. (2005). We extracted tissue sample DNA using the DNeasy Blood and Tissue kit (Qiagen, Valencia, CA) with the manufacturer's protocols. For extracted DNA samples received from the Frozen Zoo^®^, we determined the DNA concentrations using a NanoDrop Spectrophotometer (Thermo Fisher Scientific, Waltham, M.A.) and diluted to a 15 ng/µl concentration for amplification using the polymerase chain reaction (PCR).

To address whether the evolution of *MC1R* differs from the evolution of selectively neutral loci in Lorisidae, we sequenced fragments from the *MT‐CYB* and the *MC1R* (Table 2). Our *MC1R* primers were designed from Mundy and Kelly's (2003) slender loris (*Loris*) sequence (GenBank Accession: AY205137.1), but modifications to the primers were needed. We used Primer3 to design primers for successful amplification. These primers amplified a region of *MC1R* known to contribute to melanin production (171 bp–987 bp). The PCR was performed in 25 µl volumes containing 1× PCR Gold Buffer (50 mM KCL, 8 mM Tris‐HCL), 0.2 mM dNTPs, 0.4 µM each forward and reverse primers, 2 mM MgCl_2_, 1× BSA, 0.5 U AmpliTaq Gold DNA Polymerase (Thermo Fisher Scientific, Waltham, MA), and 1 µl (~15 ng) of DNA template. Amplifications were performed under the following conditions for all genes: enzyme activation at 95°C for 10 min followed by 40–45 cycles of denaturation at 95°C for 1 min, primer annealing for 1 min at locus‐specific temperatures (Table 2), and primer extension at 72°C for 1 min with a final elongation step at 72°C for 10 min and included a no‐DNA sample to detect contamination of the reagents and a positive control to detect possible failure of the PCR. Amplification products were sequenced in both directions at the University of Missouri DNA Core Facility in a 3730x1 96‐capillary DNA Analyzer with Applied Biosystems BigDye Terminator cycle sequencing chemistry (Thermo Fisher Scientific, Waltham, MA).

**TABLE 2:** Primer sequences used for this study

Sequence/base pairs	Forward primer	Reverse primer	Annealing temp	Source
MT‐CYB/331	CCA TCC AAC ATC TCA GCA TGA TGA AA	CCC TCA GAA TGA TAT TTG TCC TCA	55°C	^2^
Rag2/716	GAT TCC TGC TAY CTY CCT CCT CT	CCC ATG TTG CTT CCA AAC CAT A	55°C	^3^
	GAT TCC TGC TAY CTY CCT CCT CT	GAT AGC CCA TCC TGA AGT TCT	55°C	^2^ & ^3^
	GTG GAT TTT GAA TTT GGG TGT	CCC ATG TTG CTT CCA AAC CAT A	55°C	^2^ & ^3^
*MC1R*/729	AGT GCC TGG AGG TGT CTG T	GCA CCT CCT TGA GTG TCT TG	60°C	^1^
	AGT GCC TGG AGG TGT CTG T	AAT GAA GAG GGT GCT GGA GA	58°C	^1^
	ATA TCA CAG CAT CGT GAC TCT	GCA CCT CCT TGA GTG TCT TG	55°C	^1^

^1^ Designed by Munds using Primer3 (Rozen & Skaletsky, 1998).

^2^ Kocher et al. (1989).

^3^ Perelman et al. (2011).

### 2.5 Phylogenetic reconstruction and test of selection

The software GENEIOUS v.8.0.5 (Biomatters, Ltd.) was used to align and edit forward and reverse sequences, remove primers, and test for the presence of pseudogenes by translating sequences. We acquired outgroup sequences of *Galago*, *Eulemur*, *Varecia rubra*, *Varecia variegata*, *Callimico goeldii*, *Leontopithecus rosalia*, *Macaca nigra*, and *Macaca mulatta* from GenBank (Table 1). Besides *Galago* and *Eulemur*, the additional outgroup members were selected based on their coat color being distinctly dark (*V. variegata*, *C. goeldii*, *M. nigra*) or light (*V. rubra*, *L. rosalia*, *M. mulatta*). This provided us an opportunity to explore whether these distinct light/dark variants shared mutations found within the Lorisidae family. Outgroup sequences were aligned to our Lorisidae sequences before importing them into MEGA (Kumar et al., 2008) in FASTA format. For protein analyses, *MC1R* was translated into protein sequences using MEGA, as this study focused on the functional consequences of protein changes from *MC1R*. All aligned sequences were exported from MEGA.

*MT‐CYB* and *MC1R* sequences were individually uploaded to jModeltest ver. 2.1.7. to determine the optimal model of nucleotide substitution under the AICc criterion, which is preferred for small datasets. Based on our results from jModeltest, *MT‐CYB,* we used a general time‐reversible substitution with gamma‐distribution rates and proportion of invariable sites (GTR + G + I) fixed to 0.9705 and 0.35611, respectively. With *MC1R,* we used a GTR + G model with G fixed to 0.4966. The GTR model allows variable base frequencies with a symmetrical substitution matrix. The addition of G implies gamma‐distributed rate variation among sites, and the I influences the extent of static, unchanging sites in the dataset (Tavare, 1986). We used a Bayesian partitioned analysis for phylogenetic reconstruction using MT‐CYB with program BEAST ver.2.4.5 (Drummond et al., 2012). The program BEAST permits the user to analyze different gene sequences together, while maintaining the optimal substitution models for each gene (Drummond et al., 2012). We used the uncorrelated lognormal relaxed‐clock model and a Yule process of speciation on the tree prior, with birth rate as a gamma distribution (*α* = 0.001, *β* = 1,000). Gamma shape was exponential with a mean of 1. Calibration points for divergence time was a mean of 58 million years ago (MYA) with a standard deviation of 3.0 for the time to most recent common ancestor (TMRCA) for all primates, but with the separation of Lemuriformes at this time. The haplorhine TMRCA was 43 MYA with a standard deviation of 3.0. Finally, a TMRCA of 40 MYA with a standard deviation of 3.0 was used for the Galagidae and Lorisidae split. Dates used are based on well‐supported phylogenetic studies and the fossil record (Perelman et al., 2011; Seiffert, 2007; Yoder et al., 2001).

We ran four independent Markov chain Monte Carlo (MCMC) runs with 40 million generations with an initial 50,000 burn‐in where we sampled every 1,000, which was done for both log and tree files. Tracer ver. 1.4.1 was used to read log files and see whether the estimated sample size (ESS) met the minimum criteria of exceeding 200 for all parameters; our sampling was more than sufficient. We used TreeAnnotater ver 2.4.5 to prepare each tree file for examination. Our parameters for TreeAnnotater files were as follows: 25% burn‐in, with maximum clade credibility, and mean node heights. The four tree files were combined using LogCombiner ver. 1.5.3. The final combined tree file was viewed with FigTree ver. 1.3.1. The minimum displayed node support was 75% posterior probability (PP). For this study, we found it best to use the Bayesian trees instead of a species tree. The advantage of a species tree is it allows each gene tree to influence each other, whereas with concatenated trees a single gene can impact the entire phylogeny (Heled & Drummond, 2010; Liu & Edwards, 2009). But past studies on Lorisidae have demonstrated inconsistencies with the phylogenies resulting from a species‐tree analysis and found the concatenated tree to be more reliable (Munds et al., 2018; Pozzi et al., 2014).

A maximum‐likelihood estimate of the rate of nonsynonymous substitutions per nonsynonymous site to synonymous substitutions per synonymous site (*ω* = *dN/dS*) under a codon‐based substitution model (codeML) was done using paML v.4 (Yang, 2007). We ran a series of tests to see the effects of natural selection on *MC1R*. We used the *MT‐CYB* Bayesian partitioned analysis tree from BEAST as our input tree. Our phylogeny from the *MT‐CYB* tree agrees with past studies that found Lorisidae to represent a monophyletic family (Roos et al., 2004). The aligned *MC1R* codon FASTA file was used for the sequence file. A 729‐bp alignment of *MC1R* was used for the three analyzed genera of Lorisidae (*Loris*, *Perodicticus*, *Nycticebus*). The *N*. spp. and *N. pygmaeus* were analyzed separately as previous research found *N. pygmaeus* to have a deep divergence from *N*. spp., and even suggested they be their own genera (Munds et al., 2018; Pozzi et al., 2015), giving a total of five analyzed groups, with the outgroup (non‐Lorisidae species) as one set. We ran a total of nine models to estimate ω (Yang, 2007) (Table 3). As demonstrated, there are color differences among the genera of Lorisidae (Nekaris & Bearder, 2007) which prompted us to run clade and branch‐site models. These three models permit the researcher to select specific branches or clades to test for comparison to other groups on the phylogeny (Corso et al., 2016; Yang, 2007). For our analyses, we selected *Perodicticus* as the group to compare to other Lorisidae, because its coloration is generally lighter and less patterned from the Lorisinae. The *MC1R* substitutions were mapped onto the *MT‐CYB* phylogenetic tree using FigTree ver. 1.3.1.

**TABLE 3:** *MC1R* results of paML models tested. Ts/Tv is the transition‐to‐transversion ratios, ln L is the log likelihood ratio, *x* ^2^ is the calculated chi‐square statistic, and *df* are the degree of freedoms

Model	Ts/Tv	ln L	*x* ^2^	*df*	0.05 & 0.01 Chi value
Null (M0)	7.85368	2,313.748			
Neutral (M1)	7.99657	2,312.138	3.22	1	3.8415, 6.6349
Positive (M2)	7.99658	2,312.138	–	3	7.8147, 11.349
Beta (M7)	7.96388	2,301.379			
Beta and w (M8)	7.9639	2,301.379	–	3	7.8147, 11.349
Null branch model	8.32433	2,474.575			
Branch model	8.32435	2,474.575	0.0008	1	3.8415, 6.6349
Null clade model	6.85812	2,167.515			
Clade model	6.87288	2,167.515	0.148	1	3.8415, 6.6349

Note

Division lines separate nested testing groups.

Likelihood ratio tests (LRTs) were used to test for significant differences among models (Yang, 2007). Using the null model (M0), we examined the specific substitutions that occurred within Lorisiformes. The nonsynonymous mutations were checked for deleterious effects using PROVEAN (Protein Variation Effect Analyzer) v1.1.3 (Choi et al., 2012). This program lets us infer the functional effects of protein differences in Lorisidae *MC1R* sequences. We used PROVEAN's default threshold of −2.5 for these analyses to determine whether the several analyzed *MC1R* amino acid mutations were deleterious. To do so, PROVEAN gets amino acid mutation scores by averaging within and across clusters to generate a final score. If that score is above −2.5, then it is predicted to be a deleterious mutation (Choi et al., 2012).

**FIGURE 3:**
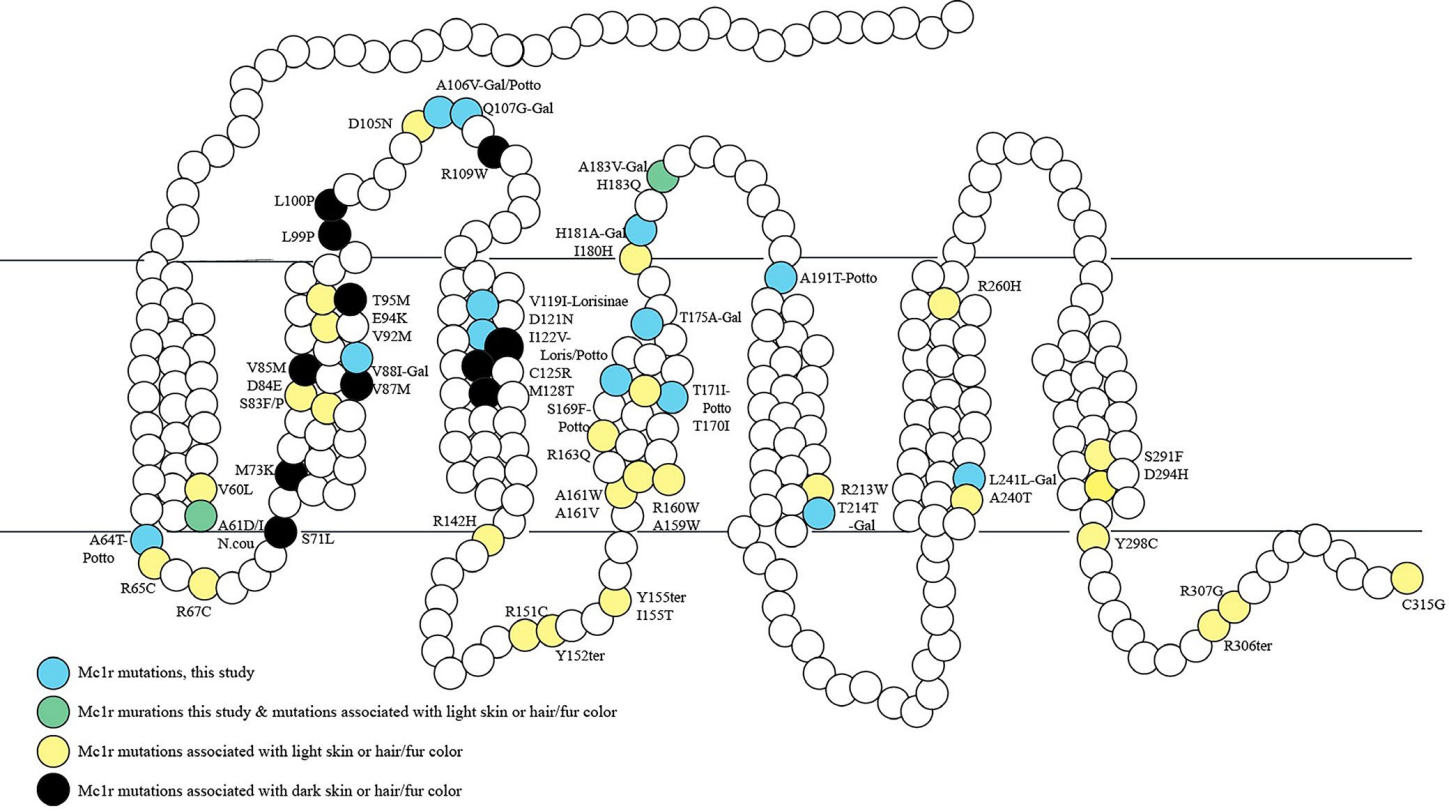
2D structure of Lorisidae *MC1R* amino acid sequence with amino acid changes indicated (blue). Mutations affecting coat color in other mammals are also indicated with either a notation for light color (yellow) or dark color (black). Circles that are green indicate both a light coloration in a vertebrate, as well as a mutation found within Lorisidae (figure modified from Buades et al., 2013)

Finally, to better understand amino acid substitutions in Lorisidae *MC1R*, we aligned our 315 *MC1R* amino acid sites to humans. This helped us determine whether mutations along Lorisidae evolution correspond to known *MC1R* mutations in other organisms. Particularly, we focused on sites known to influence color variation. Also, we examined the general location on *MC1R* (i.e., specific transmembrane, intra‐ or extracellular loop) and amino acid substitution types (i.e., aliphatic to an acid) (Figure 3).

## 3 RESULTS

### 3.1 Ancestral state reconstructions

**FIGURE 4:**
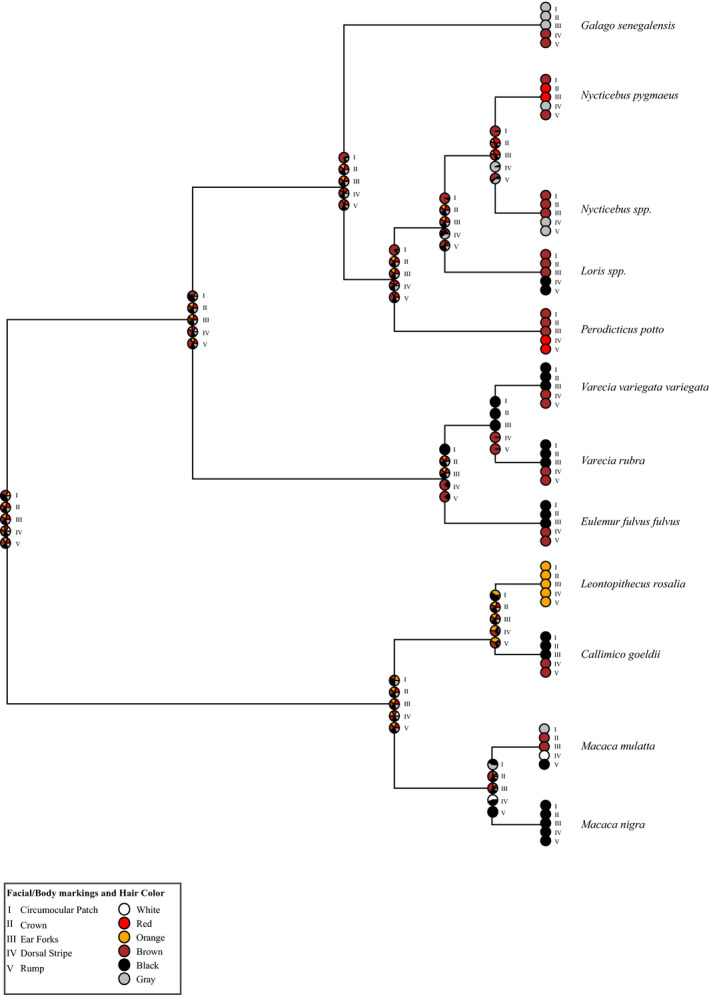
Ancestral state reconstruction of Lorisidae hair color phenotypes using 1,000 stochastic character maps under the equal‐rates (ER) model. Pie charts show the relative probabilities of each state at the internal nodes

Ancestral state reconstructions of hair color were unable to find a consensus for the hair coloration of the last common ancestor (LCA) of Lorisiformes (Figure 4); it is possible they exhibited a variety of colors, but we cannot firmly conclude such. Examining variation within strepsirrhines, lemurs, and galagos possesses a darker face (circumocular patch, crown, and ear forks), either black or gray, whereas all Lorisidae have lighter‐colored faces: brown or red. Within Lorisinae, the rump and dorsal stripes are darker in coloration: gray or black; differing from *Perodicticus potto*, which has a lighter, reddish dorsal stripe area or rump phenotype. Overall, ancestral state reconstructions of hair color demonstrate the features are highly variable, making it difficult to infer the coloration of the LCA. Yet, our results do show *Perodicticus* have a generally lighter hair phenotype in comparison to the other Lorisidae.

**FIGURE 5:**
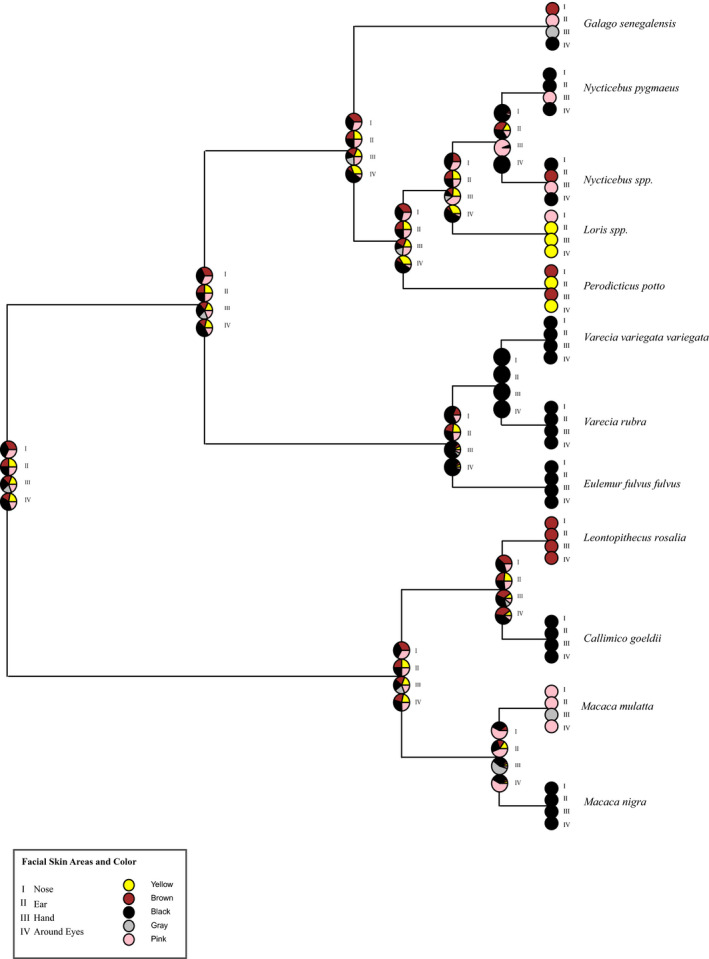
Ancestral state reconstruction of Lorisidae skin color using 1,000 stochastic character maps under the equal‐rates (ER) model. Pie charts show the relative probabilities of each state at the internal nodes

Unlike hair color, the ancestral reconstruction of the skin color of the examined primates found far less color variability, with only hand color lacking a consensus on coloration (Figure 5). These skin color traits are fairly maintained in the strepsirrhine and with the LCA of Lorisidae. Lorisiformes do stand out in comparison with the other examined primates, as most of the other primates' skin colors are monochromatic. Only Lorisiformes demonstrate differences across the features, with *Nycticebus pygmaeus* and *Nycticebus* spp., having similar coloration to *Galago*. Both *Loris* and *Perodicticus* had lighter skin color (yellow, pink, brown) for all features examined. Yet, of all Lorisidae, only *Perodicticus* had a generally lighter color appearance for hair and skin features examined.

### 3.2 Phylogenetic reconstruction and test of selection

**FIGURE 6:**
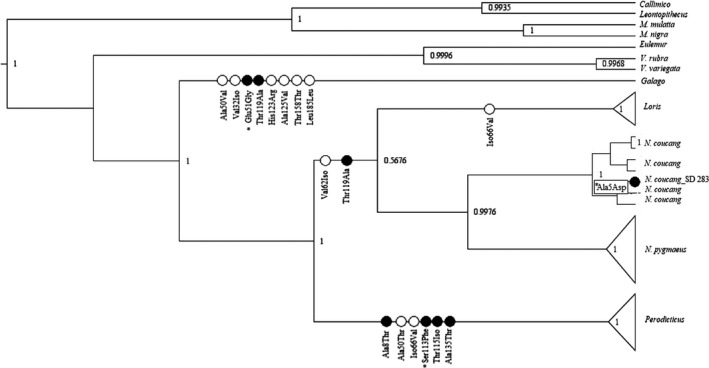
Bayesian phylogenetic tree of Lorisidae using MT‐CYB with *MC1R* nonsynonymous mutations mapped onto branches. Nonfilled circles indicate non‐charge‐changing amino acid substitutions. Filled circles are charge‐change amino acid substitutions. Site numbers correspond to specific sites found within this study's *MC1R* sequence. Those marked with a * are ones that were found to be deleterious. Node numbers are posterior probabilities

**FIGURE 7:**
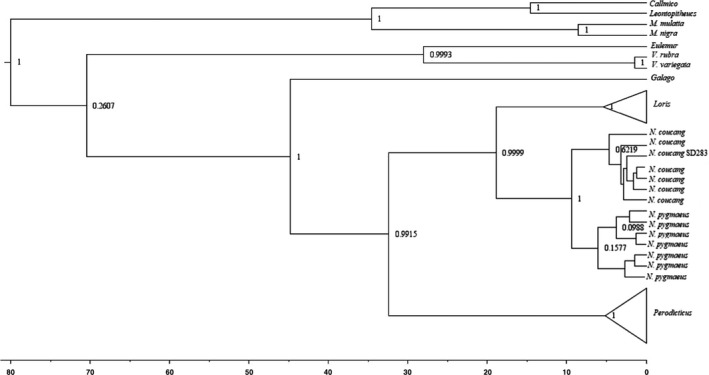
Bayesian *MC1R* tree. Node labels are posterior probabilities. Note *N. coucang* and some *N. pygmaeus* have low probability of separation using the *MC1R* phylogeny. Scale in millions of years ago

The resulting *MT‐CYB* phylogenetic tree agrees with previous studies that found Lorisidae to be a monophyletic family with Galagidae (*Galago*) as a close sister taxon (Figure 6) (Munds et al., 2018; Perelman et al., 2011; Pozzi et al., 2014, 2015). Similarly, the *MC1R* phylogeny found Lorisidae to be monophyletic (Figure 7), but unlike the *MT‐CYB* phylogeny, the *MC1R* phylogeny has a shorter time of separation between the *Nycticebus* spp. from *N. pygmaeus* (*MT‐CYB,* 21 MYA; *MC1R* 12 MYA). Our *MC1R* tree showed a long, independent evolution of *Perodicticus* from the other Lorisidae (41 MY), but the age of the *Perodicticus* genus is relatively young (6 MYO). In comparison, the Lorisinae clade (*Nycticebus*, *Loris*) arose 24 MYA, with *Loris* being 7 MYO, and *Nycticebus* being 12 MYO. Phylogenetic age results from *MC1R* are comparable to previous evolutionary history analyses of Lorisidae (Munds et al., 2018; Pozzi et al., 2015).

Of the nine models analyzed in PAML, in no cases were the more complex ones (i.e., branch or clade models) found to be significant over the simpler models (M0 and M1, Table 3). Our finding was further supported by our transition (ts)‐to‐transversion (tv) ratios, which is expected to be higher in coding genes. This is because there are fewer transversions as they are a passage of purine to pyrimidine or vice versa which result in a change of the amino acid structure. Transversions are more detrimental than transitions; transitions rarely change the amino acid as they are marked as a change from a purine to a purine or a pyrimidine to a pyrimidine. Hence, a low ts/tv ratio would imply false positives with our data, whereas a high ratio conserves the amino acid structures (Lyons & Lauring, 2017; Yang & Yoder, 1999). Although our ts/tv results do not vary much, the clade model had the lowest ratio (6.87), and the branch model the highest (8.32) (Table 3). Still, we report only the results from the neutral model (M1) and the null model (M0), which had a ts/tv of 7.99 and 7.85, respectively.

The overall ω for Lorisidae *MC1R* was 0.0912 (M0), or 96% of the proportion having a ω of 0.081 (M1), indicating purifying selection. Within Lorisiformes (*Galago* and Lorisidae), there were 75 variable sites, with only 18 nonsynonymous substitutions associated with specific branches and codons (Table 4). There were 8 nonsynonymous and 18 synonymous substitutions that led to *Galago* (Figure 6). From Galagidae to Lorisidae, there were 5 synonymous codon changes (Branch 15). Within Lorisidae, most codon changes occurred with the separation of Lorisinae from the LCA of Lorisidae (Branch 16:2 nonsynonymous, 12 synonymous) and *Perodicticus* from the LCA of Lorisidae (Branch 50:6 nonsynonymous, 12 synonymous). There were 1 nonsynonymous and 8 synonymous substitutions on branch 17 which leads to *Loris*, and there was one nonsynonymous change leading to one individual *N*. spp. (Branch 34).

**TABLE 4:** Nonsynonymous mutations in the *MC1R* locus within Lorisiformes

SITE	SEQ 1	AA 1	Type AA	SEQ 2	AA 2	Type AA	Branch	TAXON
32/88	GTG	Valine	Nonpolar	ATC	Isoleucine	Nonpolar	14	Galago
50/106	GCG	Alanine	Nonpolar	GTG	Valine	NonPolar	14	Galago
51/107	CAG	Glutamine	Polar	GGG	Glycine	Nonpolar	14	Galago
119/175	ACC	Threonine	Polar	GCT	Alanine	NonPolar	14	Galago
123/181	CAC	Histidine	Base	CGC	Arginine	Base	14	Galago
125/183	GCT	Alanine	Nonpolar	GTT	Valine	Nonpolar	14	Galago
158/214	ACC	Threonine	Polar	ACT	Threonine	Polar	14	Galago
185/241	CTC	Leucine	Nonpolar	CTG	Leucine	NonPolar	14	Galago
62/119	GTC	Valine	Nonpolar	ATC	Isoleucine	Nonpolar	16	Lorisinae
119/175	ACC	Threonine	Polar	GCC	Alanine	Nonpolar	16	Lorisinae
66/122	ATC	Isoleucine	Nonpolar	GTC	Valine	Nonpolar	17	*Loris*
5/61	GCT	Alanine	Nonpolar	GAT	Aspartic acid	Acidic	34	*N. coucang* SD 283
8/64	GCC	Alanine	Nonpolar	ACC	Threonine	Polar	50	*Perodicticus*
50/106	GCG	Alanine	Nonpolar	GTG	Valine	Nonpolar	50	*Perodicticus*
66/122	ATC	Isoleucine	Nonpolar	GTC	Valine	Nonpolar	50	*Perodicticus*
113/169	TCC	Serine	Polar	TTC	Phenylalanine	Nonpolar	50	*Perodicticus*
115/171	ACC	Threonine	Polar	ATC	Isoleucine	Nonpolar	50	*Perodicticus*
135/191	GCC	Alanine	Nonpolar	ACC	Threonine	Polar	50	*Perodicticus*

Note

Highlighted rows are charge changes. The first site number corresponds to this studies' *MC1R* sequence, and the second site number corresponds to the site on the *Homo sapiens MC1R*. Sequence (SEQ) sets are listed and their changes are listed, resulting in the specific amino acid (AA) changes.

Nonsynonymous substitutions were further explored to see whether amino acid changes involved charge changes. Out of the 18 nonsynonymous changes, 8 were associated with charge changes. The majority of these changes were from a polar to a nonpolar or vice versa (Table 4). Most charge changes were found on the *Perodicticus* branch, which had 4.

Our PROVEAN results found the majority of nonsynonymous changes were neutral. Of the examined 15 nonsynonymous changes in Lorisiformes, only 3 were determined to be deleterious (Ala5Asp, Glu51Gly, and Ser113Phe) (Table 5). As previously stated, mutations were considered deleterious if they exceed −2.5. Of the three deleterious mutations, only one mutation well‐exceeded the −2.5 threshold: Ser113Phe. This particular mutation is found on the *Perodicticus* branch.

**TABLE 5:** PROVEAN scores from *MC1R* of *Lorisiformes nonsynonymous* mutations

Variant	PROVEAN score	Prediction	Taxon
Val32Iso	−0.473	Neutral	*Galago*
Ala50Val	1.284	Neutral	*Galago*
Glu51Gly	−2.809	Deleterious	*Galago*
Thr119Ala	−0.149	Neutral	*Galago*
His123Arg	−2.039	Neutral	*Galago*
Ala125Val	3.708	Neutral	*Galago*
Val62Iso	−0.552	Neutral	Lorisinae
Thr119Ala	−0.149	Neutral	Lorisinae
Iso66Val	−0.905	Neutral	*Loris/Perodicticus*
Ala5Asp	−2.648	Deleterious	*N. coucang*, SD 283
Ala8Thr	−0.473	Neutral	*Perodicticus*
Ala52Val	1.284	Neutral	*Perodicticus*
Ser113Phe	−4.393	Deleterious	*Perodicticus*
Thr115Iso	0.359	Neutral	*Perodicticus*
Ala135Thr	0.321	Neutral	*Perodicticus*

Note

Cutoff threshold for PROVEAN scores was set at the standard ≥−2.5.

## 4 DISCUSSION

We found *MC1R* in Lorisidae is under purifying selection (*ω* = 0.0912), as predicted. Ancestral state reconstructions were inconclusive on the coloration of the LCA of Lorisidae. Phylogenetic analysis of *MC1R* found it to be semicorrelated to phylogeny based on *MT‐CYB* and previous more in‐depth phylogenetic analyses (Munds et al., 2018; Pozzi et al., 2015). The only exception was when examining the phylogenetic relationship between *Nycticebus spp*. and *N. pygmaeus*; here, *MC1R* results found the separation between the two to be shorter in depth: 21 MYO versus 12 MYO, respectively. Further examination of *MC1R* nucleotide differences found 75 variable sites associated with Lorisiformes (*Galago* and Lorisidae), but only 18 were nonsynonymous, and only 3 of these were found to be deleterious. We predicted most nucleotide site changes would be associated with the more colorful Lorisinae, but our results found that the monochromatic *Perodicticus* had the most site changes associated with their branch: 6 in total, with one being deleterious.

While our *MC1R* tree corresponds to Lorisidae phylogeny, the correspondence is not perfect. If *MC1R* were purely indicative of phylogeny, we would expect distinctions between Lorisidae genera and even species to be discernible, especially as coat colors and patterns are used to discriminate Lorisinae species (Munds et al., 2013; Nekaris & Jaffe, 2007). Alternatively, *MC1R* may not evolve fast enough to reflect recent speciation events. Because our results are based on captive individuals, we cannot confidently say *MC1R* differences are not phylogenetically based with Lorisidae, as historically captive breeding has resulted in hybrids. Our results suggest more studies should explore within species and genus variation of *MC1R*, as well as other genes known to influence pigmentation and patterning (i.e., *ASIP*, *TYR*, *TYRP1*). Such studies will help determine whether changes reflect taxonomic differences or are adaptive.

Lorisidae exhibiting purifying selection is in line with past studies examining *MC1R* evolution in other organisms, particularly in primates. *MC1R* has been found to be under purifying selection and is possibly conserved (Majerus & Mundy, 2003; Mundy & Kelly, 2003; Nunes et al., 2011; Pointer & Mundy, 2008; Shimada et al., 2009). One explanation as to why purifying selection is common with *MC1R* studies is that single nonsynonymous substitutions in *MC1R*, and similar to many other coding genes, cause large phenotypic changes that can be detrimental to the survival of an organism (Cvijovic et al., 2018; Hoekstra, 2006; Mundy & Kelly, 2003; Nunes et al., 2011; Theron et al., 2001). Purifying selection acts on coding, nuclear, and even mitochondrial genes as a way to rid the genome of deleterious mutations, particularly when such changes could have pleiotropic effects (Cvijovic et al., 2018; Hamosh et al., 2005; Popadin et al., 2013). For example, in male tawny owls (*Strix aluco*) there are two morphs (light and dark) due to melanocortin system mutations, with darker males not only having a higher survival rate but also producing higher quality, dark morph offspring. Studies found melanin‐associated mutations affected stress responses in the owls; light morphs were more susceptible to stress which impacted offspring survival and their own survival (Emaresi et al., 2014).

We expected *MC1R* nucleotide changes to be associated with the more patterned and colorful Lorisinae (*Loris* and *Nycticebus*), but that was not the case. We predicted this as researchers have speculated Lorisinae patterning and coloration are adaptive (Nekaris & Bearder, 2007; Nekaris et al., 2013, 2019). Our study was unable to truly test the relationship between venom and coloration, as only *Nycticebus* possess venom, and the genetics of venom production in this species is still being explored. We hope in the future to determine the correlation between venom and coloration in these mammals. But the results from our study do setup exploring the proximate molecular mechanisms regarding the adaptive purposes of coat colors and patterns; future research should bridge these gaps as has been done in beach and deer mice (c.f., Barrett et al., 2019; Hoekstra, 2006; Nachman et al., 2003). Only four nonsynonymous mutations occurred on Lorisinae branches, with one resulting in a charge change (Ala61Asp). The majority of nonsynonymous charge changes in Lorisidae (*n* = 6) were found on the *Perodicticus* branch. These changes appear to influence the lighter morphology of *Perodicticus*.

Regarding the amino acid substitutions of *Perodicticus*, there is one in the first transmembrane or the protein domain boundary of the first transmembrane and first intracellular loop (Ala64Thr), one on the second extracellular loop, (Ala106Val), one in the third transmembrane, which is shared with *Loris*, (Iso122Val), and one on a protein domain boundary between the fifth transmembrane and third extracellular loop (Ala191Thr) (Figure 3). Changes in the first intracellular loop of *MC1R* impact signaling activity, often as a loss of function (Wolf Horrell & D'Orazio, 2016). Yet, the most significant substitutions for *Perodicticus* occurred in the fourth transmembrane. Transmembrane regions are noted to be highly conserved and changes in these areas are often damaging (Peters et al., 2016). Furthermore, only *Perodicticus* possessed a major deleterious mutation. Although our findings are based on captive Lorisidae, of the 9 genera and 11 primate species examined only *Perodicticus* had the Ser to Phe deleterious mutation (Ser169Phe, −4.393). Other studies on *MC1R* in primates did not find the Ser to Phe mutation, but these studies did not include *Perodicticus* (Mundy & Kelly, 2003). This Ser to Phe nonconservative substitution in *MC1R* has been noted to result in a blanched, paling, or lighter phenotype in horses and fur seals (Marklund et al., 1996; Peters et al., 2016). Not only is this particular mutation associated with a lighter phenotype, but also studies have found substitutions in the fourth transmembrane result in lighter phenotypes too. Fourth transmembrane mutations in humans (R160W; R163Q; I180H) result in red hair and fair skin (Dessinioti et al., 2011; Gerstenblith et al., 2007; Pavan & Sturm, 2019; Raimondi et al., 2008). For lizards, a fourth transmembrane of Thr to Iso at 170 results in a blanched phenotype (Rosenblum et al., 2010); *Perodicticus* possess an identical Thr to Iso substitution at 171.

These nonsynonymous substitutions in *Perodicticus* are potential examples of parallel evolution: the development of a shared trait between unrelated species (Zhang & Kumar, 1997). Initially, parallel phenotypes were thought to arise from differing genetic mechanisms, but more studies are finding these phenotypes are from changes in the same genes, even across diverse taxa (Hoekstra, 2006; Miller et al., 2007). In fact, our *MC1R* study found that 5 *Perodicticus* substitutions along with 1 mutation in *Galago* (Ala183Val) are known to cause lighter coloration in other vertebrates (Table 4; Figure 3). Two of these substitutions (Ala to Val and Ala to Thr) are attributed to causing red coloration in pigs (Peters et al., 2016). The other two mutations of *Perodicticus* (Ser169Phe, Thr171Iso) could be contributing to their monochromatic, blanched phenotype (Peters et al., 2016; Rosenblum et al., 2010). Not only is the specific type of amino acid substitutions noted to cause lighter coloration in other vertebrates, but also the location of these substitutions in *MC1R* is noted to cause lighter coloration. For example, one of the more notable *Perodicticus* mutations is along the first intracellular loop (Ala64Thr). Substitutions on the first intracellular loop are known to contribute to adaptively lighter phenotypes in beach mice (Arg65Cys), mammoths (Arg67Cys), goats (Ala61Iso), and even humans (Fontanesi et al., 2006; Hoekstra, 2006; Pavan & Sturm, 2019; Peters et al., 2016). Our findings imply *MC1R* takes similar paths of evolution to produce shared phenotypes across a variety of organisms, including *Perodicticus* (Hoekstra, 2006; Miller et al., 2007).

Additionally, being a solid color with no patterns for a nocturnal, arboreal mammal is uncommon. The monochromacy of *Perodicticus* could be a result of relaxed selection due to its ecology, instead of a deleterious mutation. *Perodicticus* are known to prefer dense forest habitats and are less active under photopic light. As such, certain patterns or colors for camouflage could be unnecessary (Nekaris & Bearder, 2007). Furthermore, studies have found Perodictinae, more so than Lorisinae, communicate via olfaction (Charles‐Dominique, 1977): reducing the need for striking contrasting markings for species or mate recognition. Loss of pigmentation has been noted in cave‐dwelling creatures and examples of other vestigial traits abound in the wild (c.f. Lahti et al., 2009). For example, cichlid color diversity varies drastically depending on water clarity. Color signals in cichlids are used for sexual selection, camouflage, and species diversity (Henning et al., 2013; Seehausen et al., 1997). These ecological and behavioral preferences of *Perodicticus*, along with not being venomous or having any mimetic behavior, could be contributing to the loss of patterning and color variability in *Perodicticus*.

The contrasting patterns of Lorisinae are believed to play an important mimetic role for *Nycticebus* in mimicking cobras and for *Loris* in mimicking cobras or slow lorises (Nekaris et al., 2013). Thus, it seems purifying selection on *MC1R* in Lorisidae is to maintain these contrasting facemasks and coloration as they are potentially adaptive in Lorisinae. Although our ancestral state reconstructions were inconclusive in determining the coloration of the LCA (Figures 4 and 5), our phylogenetic results support the idea that a monochromatic appearance is derived. In many ways, maintaining striking facemasks and vibrant colors makes sense, as mammals started out as nocturnal and such patterns and coloration have been found to aid in mate recognition, predator avoidance, camouflage, and aposematic signaling (Caro, 2005; Nekaris et al., 2013). Many semiarboreal and arboreal nocturnal mammals still possess striking patterns and contrasting facemasks and dorsal stripes (i.e., felines, sugar gliders, civets) for signaling in the dim moonlight (Allen et al., 2010; Ancillotto & Mori, 2016; Caro, 2013). Losing these signals could impact survival for Lorisinae.

Yet, others have noted, *MC1R* is not strongly correlated to coat color in primates (Bradley et al., 2013; Bradley & Mundy, 2008; Haitina et al., 2007; Mundy & Kelly, 2003). It is likely that several genes are responsible for the color variation observed in Lorisidae. Ours is not the first study to come to this conclusion, and other proposed promoter genes, such as *ASIP*, *TYRP1*, solute carrier family 24 member 5 (*SLC24A5*), and dopachrome tautomerase (*DCT*) may influence coat colors but research that has examined their impact was not conclusive (Bradley et al., 2013; Haitina et al., 2007; Mundy & Kelly, 2006). More research is needed to better understand the coat color and pattern genetics of primates. Incorporating genome‐wide studies and transcriptomics will be invaluable in understanding why, of all mammals, primates are so vibrantly colored and patterned (Bradley & Mundy, 2008).

Our study found several *MC1R* amino acid substitutions that appear to influence the hair color, and possibly skin color, of Lorisidae primates. In particular, the substitutions on the *Perodicticus* branch are strongly correlated to other vertebrate studies that found similar *MC1R* substitutions to be associated with lighter hair and/or skin color. Although putative, such a finding is supported by our phenotypic analyses which demonstrate that *Perodicticus* possess a monochromatic, lighter phenotype in comparison with Lorisinae. We recognize the limitations of our study as it is difficult to definitively answer evolutionary questions based on captive populations. Yet, such research provides a window into understanding and developing future projects that can further explore this topic in‐depth. Lorisidae species are challenging to catch in the wild (Nekaris et al., 2020), but they are a fascinating family to study the evolution of coat color and patterns for aposematism (Nekaris et al., 2013, 2020). Future research on these primates and on these topics should examine the genetics of wild populations and incorporate other genes that are known to influence patterning and coloration. Furthermore, linking the genetic analyses directly to phenotypes will help determine whether the patterning and coloration are adaptive. Such work will help us better understand the colorful palette displayed by primates and ascertain the adaptive purposes behind the colors.

## CONFLICT OF INTEREST

The authors declare no conflicts of interest.

## AUTHOR CONTRIBUTIONS

**Rachel A. Munds:** Conceptualization (lead); data curation (equal); formal analysis (lead); investigation (equal); methodology (equal); writing‐original draft (lead); writing‐review & editing (equal). **Chelsea L. Titus:** Data curation (equal); funding acquisition (equal); investigation (equal); methodology (equal); writing‐original draft (equal); writing‐review & editing (equal). **Lais A. A. Moreira:** Formal analysis (equal); methodology (equal); visualization (equal); writing‐review & editing (equal). **Lori S. Eggert:** Data curation (equal); funding acquisition (equal); methodology (equal); resources (equal); supervision (equal); writing‐original draft (equal); writing‐review & editing (equal). **Gregory E. Blomquist:** Formal analysis (equal); supervision (equal); writing‐original draft (equal); writing‐review & editing (equal).

## ETHICAL APPROVAL

The authors adhered to appropriate animal care guidelines maintained by the University of Missouri. Although this study was noninvasive, we followed The Animal Behavior Society's Guidelines.

## Supporting information

Supplementary Material

Supplementary Material

## Data Availability

Sequenced genes from this study (Lorisidae *MC1R*, MT‐CYB, and Rag2) are available on GenBank. For specific identification, see Supplemental Materials 3. The photographs used in this study are the property of the American Zoological Association and cannot be made publicly available. For those interested in accessing them, please contact R. Munds, who will provide them with approval from the AZA.
