# Supplementary material for: Examining the molecular basis of coat color in a nocturnal primate family (Lorisidae)
Source: Ecol Evol. 2021 Mar 10;11(9):4442–59. doi: 10.1002/ece3.7338 (PMC8093732; doi:10.1002/ece3.7338)

**Supplemental Material 2** Examples of hair (**A**) and skin (**B**) color categories used for Ancestral State Reconstruction

**A.** Hair Color

1. White

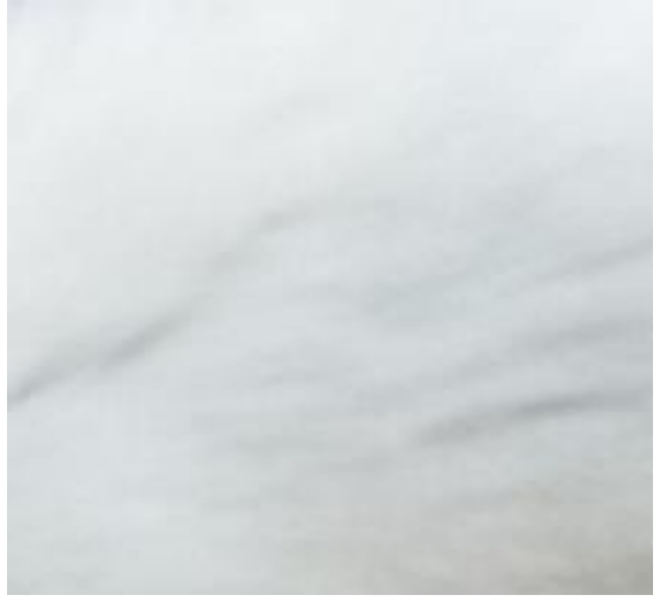

2. Yellow

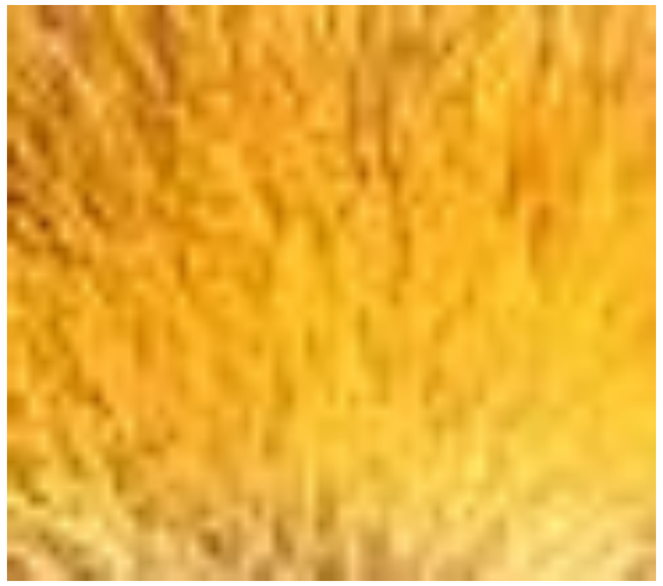

3. Red

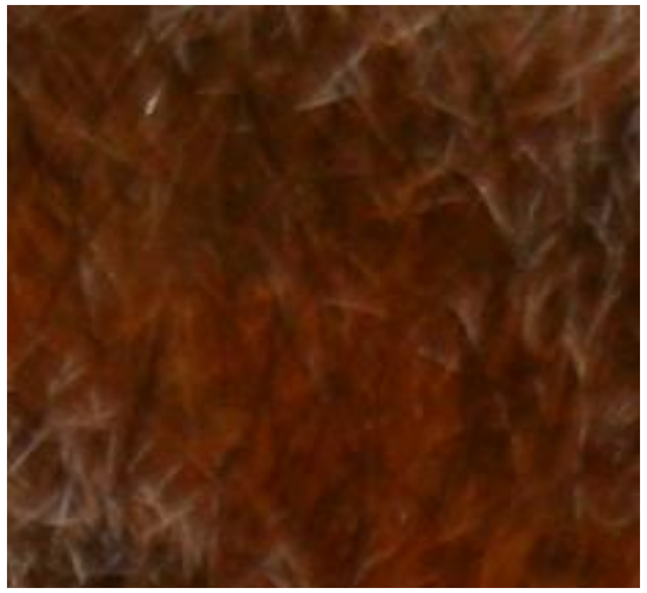

4. Orange

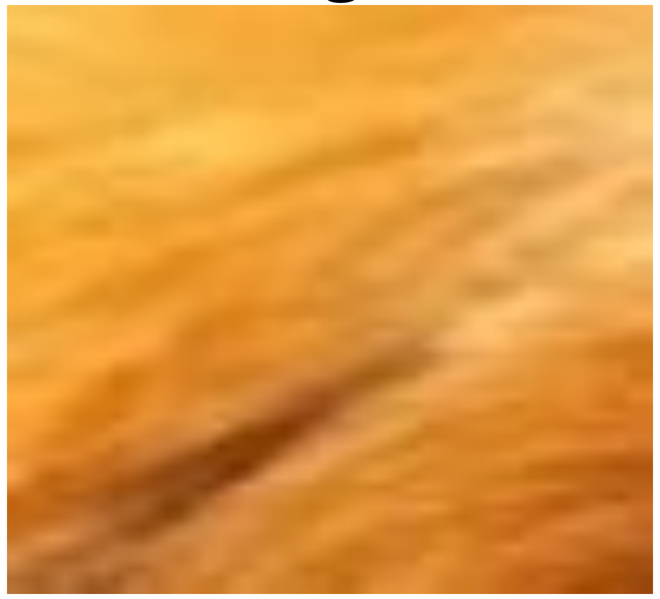

5. Brown

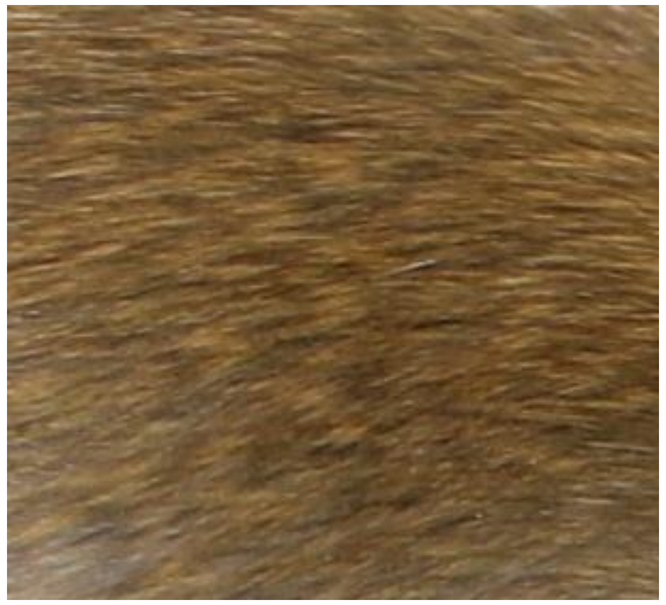

6. Black

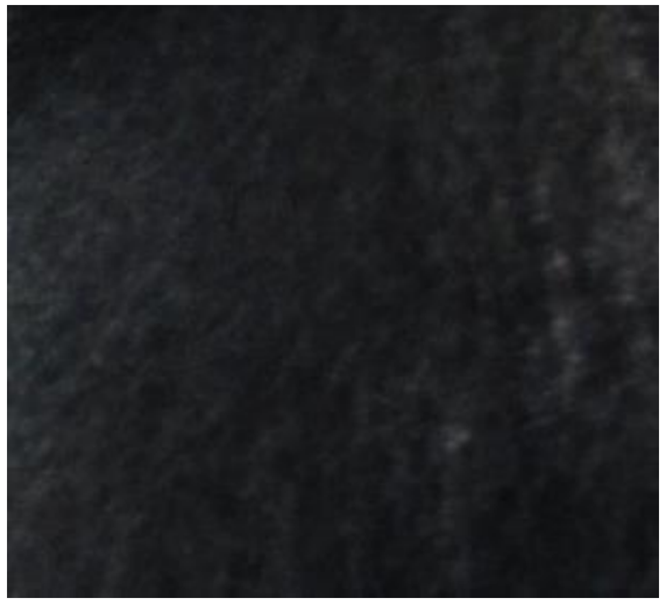

7. Gray

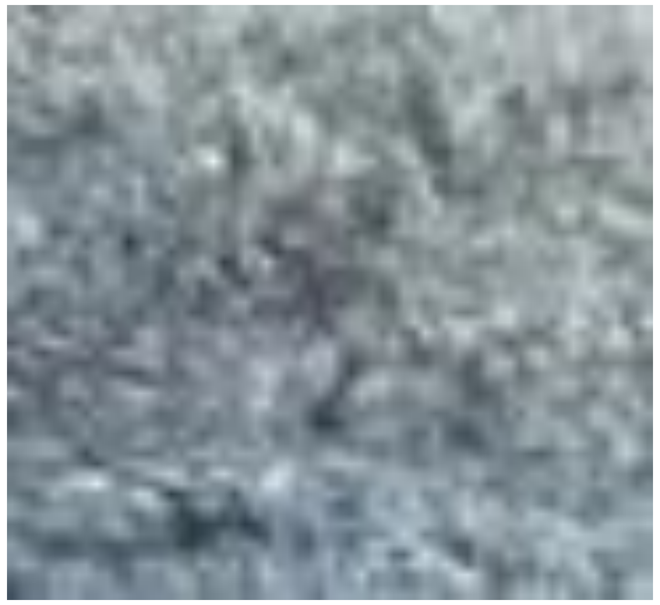

**B.** Skin Color

1. White

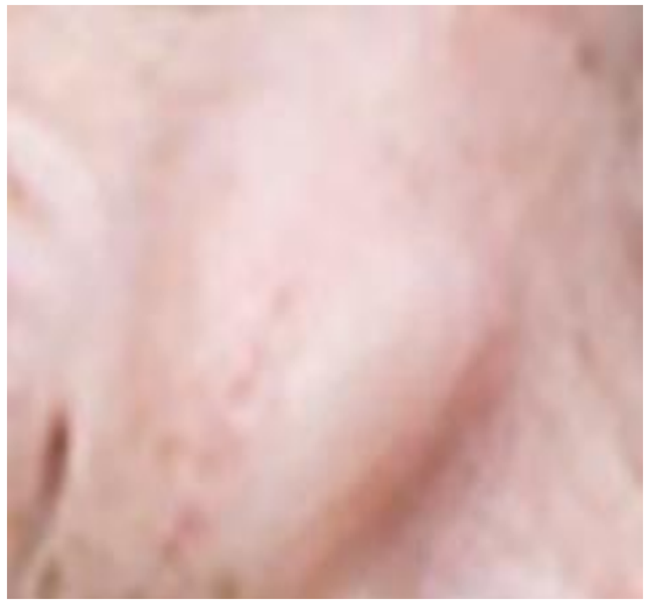

2. Yellow

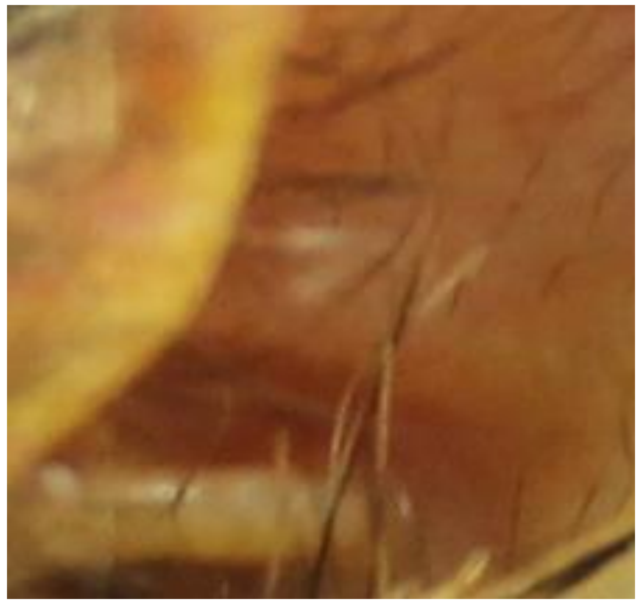

3. Red

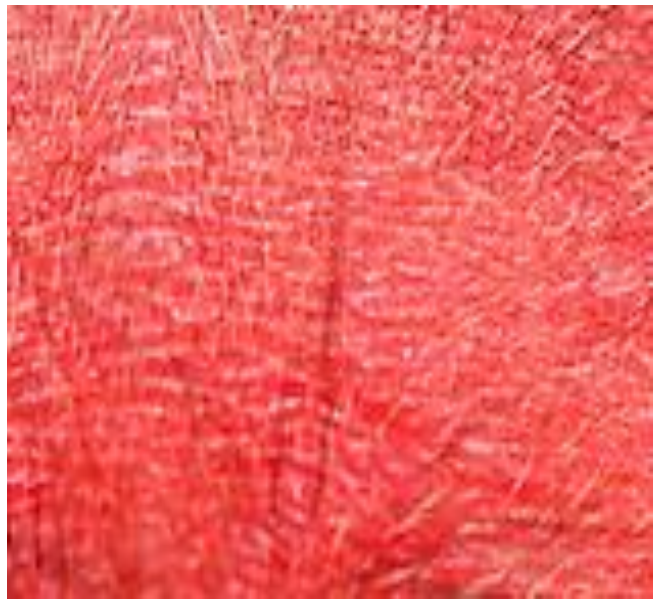

4. Pink

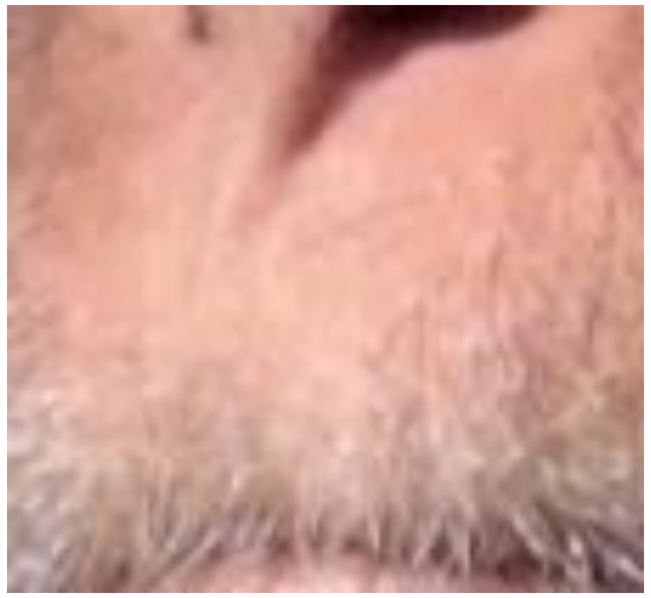

5. Brown

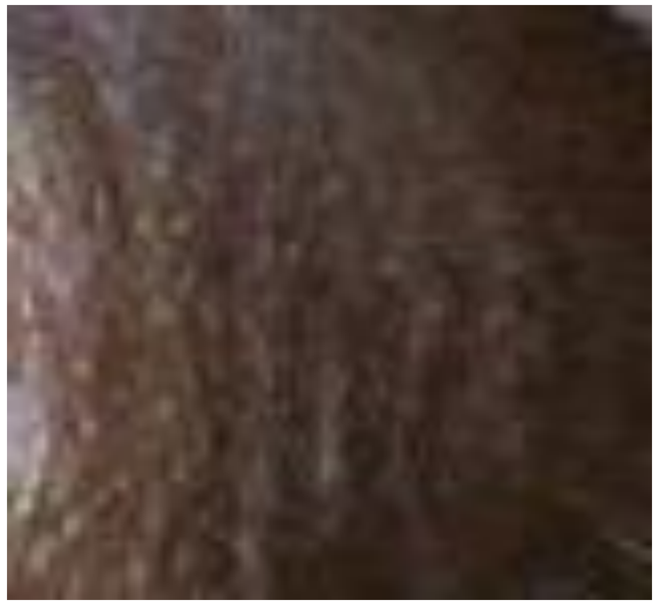

6. Black

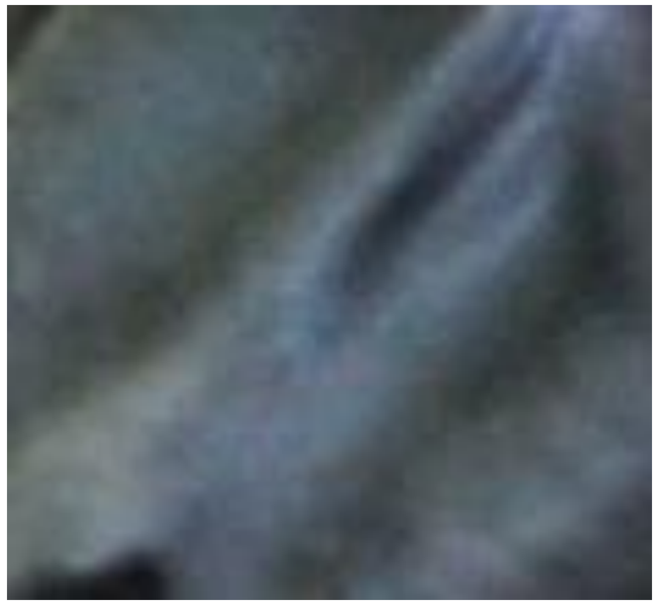

7. Gray

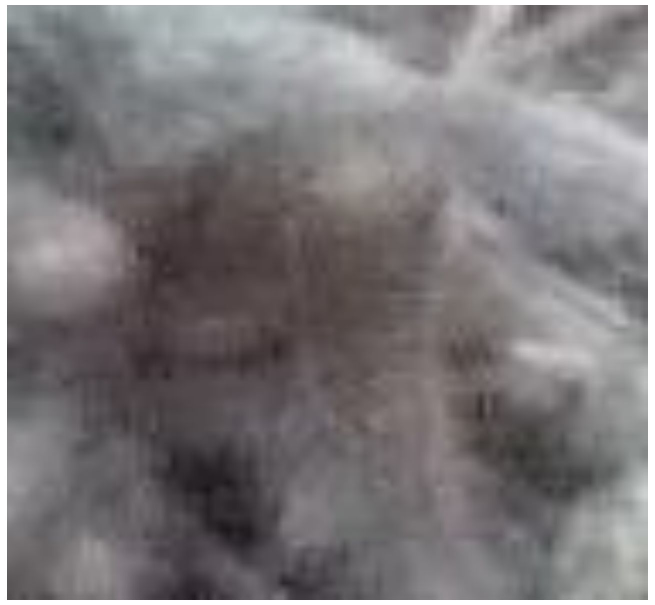

Supplement: Supplementary file 2 — Supplementary Material [file ECE3-11-4442-s001.pdf]
